# The Council Election of the English Royal College of Surgeons

**Published:** 1914-06-20

**Authors:** 


### THE PROFESSION OF MEDICINE.

### The Council Election of the English Royal College of Surgeons.

On the second of July the Fellows of the Royal College of Surgeons of England will vote for the election of five members of the Council; and there is every sign that the result of the ballot is exciting a good deal more than the average amount of interest, not to say excitement. From the point of view of the Members of the College, and particularly of those Members who have taken a prominent part in the agitation for a share in the government of the College, the election is perhaps colourless; as far as we know there is no likelihood of any of the fourteen candidates who have been nominated making this a prominent plank in his platform, or the remotest chance of success for any who should do so. Sooner or later, in our view, the "democratisation" of the College has got to come; the present exclusion of nine-tenths of the constituents from all share in the management and franchise of the body corporate is a state of affairs which cannot last indefinitely. But certainly there is no prospect of any such change being brought about in the immediate future, or of much visible progress in the movement for bringing it into being. That, however, is another issue, which is for the moment irrelevant to the subject of the forthcoming election.

It so happens that there is only one retiring member of the Council who seeks re-election—namely, Mr. C. A. Ballance, M.V.O. It does not follow that retiring members are necessarily re-elected; indeed, instances to the contrary are not at all rare. But as a general rule members retiring by rotation are certain of strong support for re-election, and are better placed on the poll than those who have not previously been elected. Mr. Ballance's chance must therefore be esteemed a very good one, all the more so because he represents a leading London medical school (St. Thomas's) and has not long ago added to his prestige by becoming Chief Surgeon to the Metropolitan Police, the last holder of which office was not only a member of the Council, but a Vice-President of the College too.

No doubt the fact that no other candidate for the five vacancies has been occupying a seat accounts for the unusual number of competitors. There are fourteen altogether, including Mr. Ballance, many of whom are prominent figures in the surgical world. There should be a very close finish between these numerous candidates when the result of the poll is declared; and it is quite likely that there are one or two surprises in store for those who fancy they can "spot" the selected five. Roughly speaking, each of the London and the leading provincial schools regards itself as morally entitled to be represented on the Council by one member. Any one which chances to be for a time unrepresented feels a certain mild sense of grievance, and the sense of *esprit de corps* among its alumni who are Fellows of the College usually leads them to vote for any presentable candidate of their old school at the next opportunity, even if they have no particular affection for him. This local patriotism is all right in a way, for it prevents any one school securing a preponderance and makes for breadth of view in the deliberations of the Council. It has, however, two drawbacks: one is that a candidate may secure election not so much on his personal merits and fitness as because his school happens at the moment to be unrepresented; and the other is that an invaluable man may fail to be elected mainly because there is already one of his colleagues on the Council. The fact that each voter may vote for only one candidate, or for two, three, four, or five, encourages this system. The "plumping" method is not in our opinion a good one; but it is in force and must be reckoned with as an important factor in deciding the elections.

Taking the candidates in the order of their names on the official list, which is that of their seniority as Fellows, the first is Mr. Ballance, whose chances have been discussed already. Next is Mr. Stanley Boyd, the well-known Charing Cross Hospital surgeon, a senior consultant whose dignified mien and eloquence would render him an ornament to any deliberative body. Next comes Mr. J. B. Lawford, ophthalmic surgeon to St. Thomas's Hospital, and surgeon to the Royal London Ophthalmic Hospital. As will presently appear, there is another candidate this year from among the specialised regional branches of surgery. Mr. Lawford is not, therefore, the only representative who will claim the votes of those who feel that the surgery of the throat, nose, ear, and eye should have representation on the Council; nor must it be forgotten that Sir John Tweedy, ex-President of the College, follows the same speciality as does Mr. Lawford, and represents it worthily.

Fourth on the list is Mr. William Thorburn, of Manchester. This well-known surgeon evidently commands the confidence of the North of England, if one may judge by the names of his three proposers, who hail from Birmingham, Newcastle, and Manchester respectively, and would be hard to equal as leaders of surgical thought in those virile centres of progress. Then we come to Mr. T. H. Openshaw, C.M.G., of the London Hospital, whose decoration was received for services with the Yeomanry Hospital during the Boer War. Mr. Openshaw had already made a mark as a surgeon before that, and is especially interested in the surgery of deformities; among other of his appointments, he is surgeon to the Royal National Orthopædic Hospital. Mr. W. G. Spencer is an old St. Bartholomew's man, who has been on the staff at the Westminster Hospital for a good many years now. He has been Jacksonian Prizeman, Arris and Gale lecturer, and Erasmus Wilson lecturer, and is also on the court of examiners of the College. The seventh and eighth in seniority are Mr. Raymond Johnson, surgeon to University College Hospital, and Mr. F. F. Burghard, a Guy's man, who is surgeon to King's College Hospital.

There is not much profit in attempting to distinguish between the seniors and juniors of the competing fourteen; but if any such line is to be drawn, it would probably divide the foregoing, who have all been Fellows for a quarter of a century or more, from the remaining six, whose Fellowships fall within the period 1891 to 1898. The first of these, ninth on the complete list, is Mr. T. H. Kellock, an old St. Thomas's man, whose staff appointments are at the Middlesex and Great Ormond Street Hospitals. Then come Mr. McAdam Eccles, of St. Bartholomew's, well known for energetic support of total abstinence; and Mr. Macleod Yearsley, a London and Westminster Hospitals man, and one of the leading authorities on aural surgery. Next is Mr. Charles Ryall, another old Westminster student now on the staffs of the Cancer and the Lock Hospitals. He is quite likely to prove one of the " dark horses " of the election, partly from his personal popularity and partly as a representative of the special hospitals other than those concerned with eyes, ears, noses, and throats. Thirteenth in seniority is Mr. H. S. Pendlebury, surgeon to St. George's and to the Royal Waterloo Hospitals. He has been intimately connected with the foundation and management of the Royal Society of Medicine, which may bring him votes. Last of all is Mr. F. J. Steward, surgeon to Guy's Hospital and formerly also at Great Ormond Street. He is joint author with Mr. Jacobson of the " Operations of Surgery," which the latter originated.

It will thus be apparent that the electors have a varied choice from among a most distinguished band of competitors, thirteen of whom are resident in London and only one in the provinces. The ballot will be open from 3 to 5 p.m., and the issue of it will certainly be awaited with more than the customary curiosity. There is an impression about in Harley Street that some of the candidates have carried their canvassing to an undignified point. But then this method of gaining support is not forbidden, nor is it contrary to the custom of the College; how these more-energetic-than-usual candidates will fare at the poll remains to be seen.

